# Associations of high-risk drug patterns with mortality among community-dwelling older adults: A 23-year prospective cohort study

**DOI:** 10.1371/journal.pone.0332210

**Published:** 2025-09-11

**Authors:** Liat Orenstein, Angela Chetrit, Ronen Fluss, Keren Laufer, Moyses Szklo, Rachel Dankner

**Affiliations:** 1 Public Health Research Center, Gertner Institute for Epidemiology and Health Policy Research, Sheba Medical Center, Ramat-Gan, Israel; 2 Department of Epidemiology and Preventive Medicine, School of Public Health, Gray Faculty of Medical and Health Sciences, Tel-Aviv University, Tel-Aviv, Israel; 3 Biostatistics and Biomathematics Unit, Gertner Institute for Epidemiology and Health Policy Research, Sheba Medical Center, Ramat-Gan, Israel; 4 Pharmaceutics Registration Department, Teva Israel Ltd., Shoham, Israel; 5 Department of Epidemiology, the Johns Hopkins Bloomberg School of Public Health, Baltimore, Maryland, United States of America; Livingstone Center for Prevention and Translational Science, ZAMBIA

## Abstract

**Background:**

Data on drug safety in multimorbid older-adults are limited, as clinical trials often apply upper age limits and focus on individual drugs or specific combinations. We aimed to explore high-risk drug patterns in community-dwelling older-adults, and their associations with long-term mortality.

**Methods:**

We included 1,048 participants from a longitudinal population-based cohort, all taking at least one medication. Participants were examined in 1999–2007 and followed for mortality through March 2022. Individuals with similar profiles of high-risk drugs, identified using Beers criteria as potentially inappropriate for most older adults or requiring caution, were grouped using agglomerative hierarchical clustering. Cox and competing-risk regressions were used to examine the associations of the high-risk drug patterns with all-cause and non-cancer mortality.

**Results:**

The most prevalent morbidities among participants (mean age 73.3 ± 7.3 years, 55.9% women) were hypertension (55.3%) and cardiovascular diseases (45.5%), and 77.7% took at least one high-risk drug. Five distinct patterns were identified: ‘None’ cluster (no dominant high-risk drug); ‘Calcium channel blockers’ (CCBs) cluster, with high nonsteroidal anti-inflammatory drug (NSAID) prevalence; ‘Renin-angiotensin-aldosterone system (RAAS) inhibitors’ cluster, with a high concomitant use of sulfonylureas compared to other clusters; ’Diuretics’ cluster, with a relatively high prevalence of antithrombotics and proton pump inhibitors; and ’Benzodiazepines’ cluster, with a relatively high antidepressant prevalence. Clusters differed by age, sex, ethnicity, and health characteristics. In multivariable analysis, the ‘Diuretics’ cluster was associated with increased all-cause (HR = 1.33, 95%CI: 1.03–1.72) and non-cancer (HR = 1.41, 95%CI: 1.03–1.93) mortality compared to the ‘None’ cluster. The ‘CCBs’ cluster was associated with a greater risk for non-cancer mortality. Several drug combinations were identified as potential contributors to the increased risk observed in these clusters, including the concomitant use of NSAIDs and antihypertensives and a possible CCB-diuretic prescribing cascade.

**Conclusions:**

Examining high-risk drug patterns offers a patient-centered approach to improving evidence-based medication guidelines and facilitating early interventions for vulnerable older-adults.

## Introduction

Older adults often present with multiple chronic conditions [1]. As most clinical guidelines are single-disease based, multimorbidity leads to long-term treatment with multiple drugs [2]. However, high-quality data on drug safety and efficacy for this growing population are limited, largely due to their frequent exclusion from clinical trials [3–5]. Thus, guidelines often disregard the potentially different pharmacokinetics and pharmacodynamics due to age-related alterations in body composition and metabolism, the greater heterogeneity in terms of physiological reserves, and the complexities of treatment within the context of multimorbidity and polypharmacy [6,7]. Older adults are consequently more susceptible to adverse drug reactions (ADRs) than younger individuals [6,8]. Recognizing this public health concern, various tools have been developed to identify potentially inappropriate medications (PIMs) in older persons aged 65 or above, i.e., drugs that are ineffective or pose unnecessary risks [9]. The Beers criteria, a widely accepted tool, that is periodically updated with the American Geriatrics Society [10–13], classify drugs to avoid or be used with caution in older adults. Some of these drugs are associated with high rates of hospitalization (e.g., antipsychotics [14–16], antidepressants [14,16], nonsteroidal anti-inflammatory drugs (NSAIDs) [14,17], diuretics [14,18], benzodiazepines (BZDs) [14,16,19], opioids [15,20] and anticholinergics [21,22]). Additional drugs, e.g., calcium channel blockers (CCBs), renin-angiotensin-aldosterone system (RAAS) inhibitors and antithrombotics, were identified in the literature as “high risk” owing to their consistent association with increased adverse events in older adults [6,14,18,23].

The impact of drug regimens on mortality has been less studied. Some drugs are consistently associated with increased mortality risk (e.g., anticholinergics [21,24] and opioids [15,25]), whereas results vary for other drugs, such as antipsychotics [15] and BZDs [24,26]. Conversely, drugs such as CCBs [27] and RAAS inhibitors [28] are associated with decreased mortality.

Older multimorbid patients often use various high-risk drug combinations, yet limited literature exists on these patterns [20,29], and their associations with mortality [23]. In addition, widely used administrative databases do not capture over-the-counter (OTC) drugs, such as NSAIDs [15], which can interact with prescribed drugs and impact safety and adherence [30,31]. Moreover, self-medication has been associated with increased risk of drug interactions [32].

As the population ages, high-quality data are crucial for developing evidence-based medication guidelines for older adults. This study aimed to examine patterns of high-risk drug use, considering both prescription and non-prescription regimens, in a population-based cohort of community-dwelling older adults; and to further investigate their associations with all-cause and non-cancer mortality. Taking a patient-centered approach, we evaluated the combined use of high-risk medications rather than focusing on individual drugs, to better reflect real-world use and identify profiles of high-risk patients who could benefit from interventions targeting their medication use.

## Methods

### Study population

We used data from Phase 3 of the Israel Study of Glucose Intolerance, Obesity and Hypertension (GOH Study), a longitudinal study that began in 1969, with a sample of 5,711 Jewish participants, stratified by sex, age and ethnic origin. The current study is based on data collected during Phase 3 (January 1999 through November 2007), the only wave in which detailed medication information was collected. The final study cohort included 1,048 participants who were approached for the third follow-up, excluding those not taking any medications (n = 162). Face-to-face interviews covered sociodemographic characteristics, lifestyle habits and medical history; anthropometric measurements and blood samples for biochemical and genetic biomarkers were taken. The study was approved by the local IRB of the Chaim Sheba Medical Center (Approval # SMC-8394-21). All participants signed a written informed consent form upon recruitment to the original GOH Study. Since only anonymous data was used in the current study and patient privacy was not violated, obtaining informed consent for the current study was waived by the IRB (decision made on August 5, 2021).

### High-risk medications

During the baseline interview, participants presented the packaging of all medications they regularly take. Drug information (name, indication, dosage, duration) was cross-checked against a summary letter from their physician, then recorded and coded using the Anatomical Therapeutic Chemical (ATC) classification. ATC codes were assigned based on brand names, following several data-cleaning steps to ensure accuracy. Any missing or inconsistent codes were resolved in consultation with the study pharmacist (K.L.). “High-risk” medications were identified according to the 2019 Beers criteria (medications that are potentially inappropriate in most older adults, and medications that should be used with caution), which was the most updated version at that time [12], as well as from the literature [6,14,18,23]. Drugs were then grouped into 19 categories and coded as binary variables indicating whether they were taken. To ensure statistical power, only drug categories taken by at least 2% of participants were considered, resulting in 14 categories: NSAIDs, RAAS inhibitors, CCBs, diuretics, BZDs, sulfonylureas, proton pump inhibitors (PPIs), antithrombotics, antidepressants, peripheral alpha‐1 blockers, opioids, other central nervous system (CNS) drugs, antipsychotics and other anticholinergics (see S1 Table for further details).

Polypharmacy and hyper-polypharmacy were defined as the concomitant use of at least 5 or 10 drugs, respectively, including OTC drugs, vitamins and dietary supplements. This approach was chosen because, although commonly perceived as safe, these products can interact with prescription drugs, potentially altering their efficacy and safety [33,34]. Moreover, their inappropriate use (e.g., excessive dosing, prolonged use, or use for unsuitable indications) may also cause harm [35].

### Outcome measures

The study file was linked to the national mortality registry held by the Israeli Ministry of Health using a unique identification number. Linkage was performed via TIMNA (Hebrew acronym for ‘Research Infrastructure for Big Data Studies’), a national research platform established by the Israeli government to enable big-data studies combining health data from multiple organizations. TIMNA personnel merged dates and causes of death, updated through 07 March 2022, into the study data file. They then removed all identifying fields, conducted thorough de-identification, including processing quasi-identifiers, and ensured compliance with k-anonymity standards. Following this process, we were granted accessed to the complete, de-identified dataset on 7 April 2022 at 11:30am for analysis. Cancer-related deaths were identified using relevant ICD-10 codes, which are used by the Israel Central Bureau of Statistics for official cause-of-death classification based on death notifications. For each participant, follow-up began at the date of examination and ended at death or the end of follow-up, whichever occurred first.

### Potential confounders

The sociodemographic characteristics included age (continuous or age tertiles: < 69, 69–75, > 75 years), sex (men, women), years of education (continuous), ethnic origin (defined according to the reported country of birth, or the father’s country of birth for Israeli born individuals; categorized according to 4 main ethnic groups in Israel: Europe/America, Middle-East, North Africa, Yemen [36]), marital status (single, married, divorced/separated, widowed), occupation (categorized as Blue collar (manual and skilled trade occupations, including agriculture, fishing, craft, manufacturing, repair, construction, machine operation, transport, cleaning, packaging and general labor workers), lower White collar (administrative and clerical workers), and upper White collar (scientific and academic professionals, senior managers, freelancers and technical professionals)), and health maintenance organization (HMO) membership.

Lifestyle habits included leisure-time physical activity (any, none) and smoking status (current, past, never). Health factors included waist circumference (cm), body mass index (BMI, kg/m^2^; categorized according to the World Health Organization (WHO) criteria [37] as normal (<25), overweight (25–29.9) and obese (≥30)), self-rated health (excellent, very good, good, fair, poor), apolipoprotein E (ApoE) genotype (ApoE ε2 group includes ε2/2 and ε2/3; ApoE ε3 group includes ε3/3; ApoE ε4 group includes ε4/4 and ε3/4; and ‘Other’ group includes the rare ε2/4 or missing observations), and number of comorbidities (continuous). Information on chronic conditions was derived from the baseline interview. Participants were asked about diagnosed chronic conditions as well as past procedures and hospitalizations over the preceding 10 years. Conditions of interest were cancer, heart disease, lung disease, joint diseases, kidney diseases, diabetes, eye disease, gastrointestinal disease, and hypertension. This was supplemented by linkage with national hospitalization records, capturing all hospitalizations (diagnoses and procedures) prior to the baseline interview. Three conditions known as major mortality risk factors were considered individually: diabetes, cardiovascular disease and cancer.

### Statistical analysis

Building upon the methodology proposed by Huang et al. [23], the analysis proceeded in two steps. First, we identified patterns of high-risk drug use in our cohort. Next, we examined whether any of these patterns were associated with an increased risk for all-cause or non-cancer mortality.

#### High-risk drug patterns.

Hierarchical agglomerative clustering with Ward’s method was carried out to group individuals into clusters containing similar profiles of the drug categories [23]. We selected hierarchical agglomerative clustering because it is well suited for small datasets, handles binary variables effectively [38], and is particularly appropriate when the objects being classified follow an underlying structured pattern [39], such as drug co-administration for specific conditions or comorbidities, as well as contraindications. To ensure cluster stability, only drug categories with a prevalence of 10% or more were considered in the clustering process. The optimal number of clusters was determined using dendrogram visualization, the Elbow plot (evaluating up to ten clusters), statistical power considerations for cluster sample sizes, and cluster stability as assessed by computing the mean Jaccard similarity index for each cluster over 1,000 repeated random subsets of the cohort using the clusterboot function from the fpc R package. A Jaccard index score > 0.5 indicates a reproducible and stable cluster [40,41]. The ultimate goal was to derive clear and clinically valuable drug patterns that exhibit differences in mortality [23,42]. Differences between clusters in baseline characteristics were tested using the Chi-Square test for categorical variables and the Kruskal–Wallis test for continuous variables.

#### Associations with mortality.

For both all-cause and non-cancer mortality, the number of events and unadjusted event rates per 1,000 person-years, along with 95% confidence intervals, were calculated overall and stratified by cluster.

Multivariable Cox proportional hazards regression models were used to examine associations of high-risk drug patterns with all-cause mortality. Since cancer incidence and mortality is rarely drug-related [43], an additional approach focused on death from non-cancer causes. Fine-Gray subdistribution hazard models were employed to account for cancer mortality as a competing event [44]. This method is based on the cumulative incidence function (CIF), which models the probability of the event of interest occurring in the presence of competing risks. That is, individuals who died from cancer (a competing event) remained in the risk set but were weighted differently to reflect that they could no longer experience the event of interest.

For each model, the proportional hazards assumption was assessed by conducting a test for the proportionality of the interaction between time and the variables in each model. If violations were identified, interactions between time and the relevant variables were incorporated into the model. All baseline variables were initially entered into the model, and removed sequentially using backward elimination, until only those with p < 0.10 or known confounders remained. Multicolinearity was evaluated by Cramér’s V statistic and IVF/Tolerance statistics. The Akaike’s information criterion (AIC) was used to select between alternative models.

Six sensitivity analyses were employed: (1) adjusting for individual cardiovascular-related diseases; (2) adjusting for pack years of smoking and time since quitting instead of smoking status; (3) considering only the pre-COVID-19 follow-up period, to address potential bias from pandemic-related changes in health-service utilization; (4) additionally adjusting for birth cohort to address time-related factors affecting drug exposure or its association with mortality; (5) considering an alternative definition for polypharmacy, excluding vitamins and dietary supplements; and (6) performing a multiple imputation analysis. Anthropometrics and ApoE genotyping were missing for up to 19.8% of participants, primarily because some completed the third follow-up by phone. We hypothesize that those who refused/could not arrive at the regional medical clinics were in poorer health, and therefore the data were missing not at random (MNAR). This is supported by the observation that participants with missing data were generally older, less physically active, less educated, had poorer self-rated health and a larger number of comorbidities (see S2 Table). In general, multiple imputation is not recommended when data are MNAR, as it can lead to biased estimates [45,46]. To minimize this risk and preserve sample size, we used the missing-indicator approach in the main analysis. Nevertheless, we conducted a sensitivity analysis using multiple imputation under the control-based pattern assumption [47]. Further details on the sensitivity analyses are provided in S1 Text.

Statistical analyses were conducted using SAS version 9.4 (SAS Institute, Cary, NC) and R version 4.2.2 (R Foundation for Statistical Computing) software.

## Results

### High-risk drug patterns

In our cohort of 1,048 community-dwelling older adults who were taking one or more medications, 814 (77.7%) took at least one high-risk drug. The most frequently used high-risk drugs were NSAIDs (reported by 43.0% of participants), followed by RAAS inhibitors (31.2%), CCBs (25.0%), diuretics (20.9%) and BZDs (17.3%). The prevalence of other high-risk drugs was less than 10% (S1 Table).

**Fig 1:**
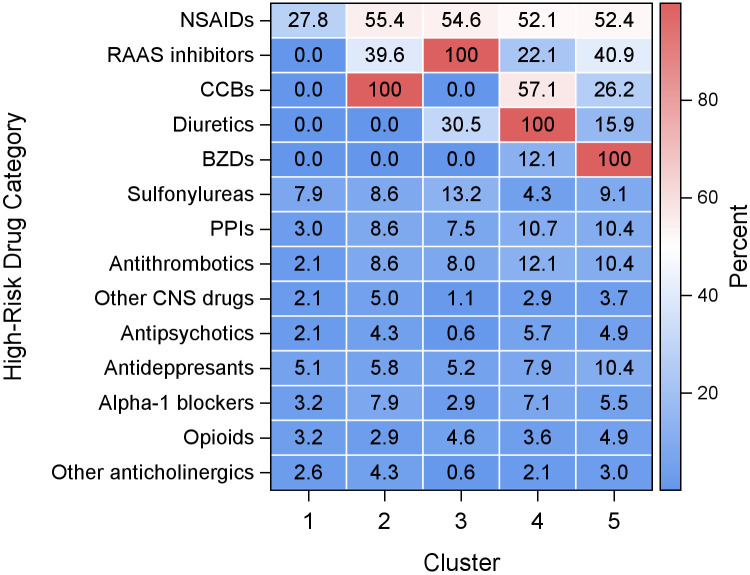
Heatmap of prevalence of 14 high-risk medication categories by cluster. Alpha-1 blockers, Peripheral alpha-1 blockers unless for benign prostatic hyperplasia; BZDs, Benzodiazepines (including ‘Z-drugs’); CCBs, Calcium channel blockers; NSAIDs, Nonsteroidal anti-inflammatory drugs; PPIs, Proton-pump inhibitors (use for >8 weeks unless high‐risk patients); RAAS, Renin angiotensin-aldosterone system. Cluster 1 = ‘None’ cluster (n = 431); Cluster 2 = ‘Calcium channel blockers (CCBs)’ cluster (n = 139); Cluster 3 = ‘Renin-angiotensin-aldosterone system (RAAS) inhibitors’ cluster (n = 174); Cluster 4 = ‘Diuretics’ cluster (n = 140); Cluster 5 = ‘Benzodiazepines (BZDs)’ cluster (n = 164).

The resolution of five clusters (i.e., high-risk drug patterns) fit our data best (see S1 and S2 Figs for the dendrogram and Elbow plot, respectively): in **Cluster 1** (n = 431) the most frequently taken drug, NSAIDs, was reported by less than one third (27.8%). This cluster also included all participants not taking any high-risk drugs, and was thus labeled ‘None’. Each of the other clusters had one drug category taken by all participants, which was used for labeling. **Cluster 2** (‘CCBs’ cluster, n = 139) had a high prevalence of NSAIDs (55.4%, compared with 27.8%−54.6% in other clusters). Compared to the other clusters, there was also a high prevalence of peripheral alpha‐1 blockers (7.9%) and other CNS drugs (5.0%). **Cluster 3** (‘RAAS inhibitors’ cluster, n = 174) had a high prevalence of sulfonylureas (13.2%, compared with 4.3%−9.1% in other clusters), and 54.6% of its participants were also taking NSAIDs. In **Cluster 4** (‘Diuretics’ cluster, n = 140), 57.1% also took a CCB. This cluster also had a relatively high prevalence of antithrombotics (12.1%), PPIs (10.7%) and antipsychotics (5.7%). **Cluster 5** (‘BZDs’ cluster, n = 164) also had a high prevalence of antidepressant use (10.4%, compared to 5.1%−7.9% in other clusters). The prevalence of all 14 high-risk drug categories across clusters is presented in Fig 1.

Table 1 presents baseline characteristics of the participants overall and by cluster. The mean age at baseline was 73.3 ± 7.3 years, with 56% women. The ‘None’ cluster included the youngest participants (mean age 71.6 ± 7.2), had the lowest mean BMI (27.2 ± 4.2), and the highest proportion engaged in physical activity (54.8%). Its participants also had the fewest comorbidities (a mean of 2.0 ± 1.5), the highest self-rated excellent health (11.1%, compared with 1.5%−5.4% in other clusters), and only 17.4% were exposed to polypharmacy. The ‘CCBs’ cluster had the highest prevalence of hypertension (90.6%) and cardiovascular disease (61.2%). The ‘RAAS inhibitors’ cluster was the only cluster with a majority of men (54.0%), and had the highest prevalence of diabetes (39.7%). The ‘Diuretics’ cluster had the oldest participants (mean age 76.3 ± 7.1), the highest proportion of participants reporting fair/poor self-rated health (39.0%), not engaging in any leisure-time physical activity (61.4%), and the highest mean number of comorbidities (3.8 ± 1.8), more specifically joint disease (34.3%), lung disease (25.0) and kidney disease (22.9%). This cluster also had the highest prevalence of polypharmacy (75.7%). The ‘BZDs’ cluster included relatively older participants (mean age 75.0 ± 7.0), with nearly two-thirds women. This cluster had the highest prevalence of gastrointestinal disease (53.0%) across clusters, and the second-highest polypharmacy prevalence (67.7%). Sociodemographic characteristics were similar across the clusters.

**Table 1:** Baseline characteristics of 1,048 community-dwelling older adults by cluster.

	Overall		Cluster 1 None (*n = *431)	Cluster 2 CCBs (*n* = 139)	Cluster 3 RAASi (*n* = 174)	Cluster 4 Diuretics (*n* = 140)	Cluster 5 BZDs (*n* = 164)	P-value
	n	%/ Mean±SD	%/ Mean±SD	%/ Mean±SD	%/ Mean±SD	%/ Mean±SD	%/ Mean±SD	
**Age (years)**	1,048	73.3 ± 7.3	71.6 ± 7.2	73.6 ± 7.3	73.4 ± 7.3	76.3 ± 7.1	75.0 ± 7.0	<0.001
**Sex**								0.007
Men	462	44.1	42.7	48.2	54.0	42.9	34.8	
Women	586	55.9	57.3	51.8	46.0	57.1	65.2	
**Education (years),** median [Q1,Q3]	1,041	10.0 [7.0, 12.0]	11.0 [8.0, 12.0]	10.0 [6.0, 12.0)]	12.0 [7.0, 12.5]	10.0 [6.0, 12.0]	10.0 [6.0, 12.0]	0.057
**Origin**								<0.001
Yemen	204	19.5	23.7	14.4	23.0	17.1	11.0	
Middle East	271	25.9	29.0	28.8	19.0	24.3	23.8	
North-Africa	186	17.7	16.9	13.7	23.0	19.3	16.5	
Europe-America	387	36.9	30.4	43.2	35.1	39.3	48.8	
**Occupation** ^a^								0.45
Upper White collar	404	38.6	39.4	36.7	42.0	37.9	35.2	
Lower White collar	197	18.8	17.9	18.0	20.1	24.3	16.0	
Blue collar	445	42.5	42.7	45.3	37.9	37.9	48.8	
**Marital status**								0.020
Married	728	69.4	72.6	69.8	71.3	68.6	59.8	
Divorced\Separated\Single	48	4.6	5.6	3.6	5.2	4.3	2.4	
Widowed	272	26.0	21.8	26.6	23.6	27.1	37.8	
**Waist circumference (cm)**	869	99.6 ± 12.0	96.5 ± 11.2	103.2 ± 12.5	101.9 ± 11.8	102.2 ± 11.2	99.8 ± 12.5	<0.001
**BMI (kg/m**^**2**^)	904	28.3 ± 4.6	27.2 ± 4.2	28.9 ± 4.3	29.5 ± 5.1	29.3 ± 4.6	28.6 ± 5.0	<0.001
**Smoking**								0.15
Never	619	59.1	63.3	54.0	53.4	61.4	56.1	
Past	336	32.1	28.1	36.0	36.2	34.3	32.9	
Current	93	8.9	8.6	10.1	10.3	4.3	11.0	
**ApoE genotype** ^b^								0.39
ApoE ε2	92	8.8	9.3	10.1	5.7	8.6	9.8	
ApoE ε3	618	59.0	61.9	56.1	61.5	57.9	51.8	
ApoE ε4	107	10.2	10.0	7.9	12.6	10.0	10.4	
Other/missing	231	22.0	18.8	25.9	20.1	23.6	28.0	
**Any sports related physical activity (yes),** %	506	48.3	54.8	41.7	51.1	38.6	42.1	0.001
**No. of comorbidities** ^c^	1,048	2.9 ± 1.8	2.0 ± 1.5	3.3 ± 1.5	3.3 ± 1.7	3.8 ± 1.8	3.4 ± 1.9	<0.001
**Diabetes (yes)**	286	27.3	19.7	28.8	39.7	30.7	29.9	<0.001
**Hypertension (yes)**	580	55.3	20.2	90.6	85.6	83.6	61.6	<0.001
**Cardiovascular Disease (yes)**	477	45.5	29.5	61.2	51.1	58.6	57.3	<0.001
**Joint Disease (yes)**	267	25.5	20.6	22.3	25.3	34.3	33.5	0.002
**Lung Disease (yes)**	169	16.1	13.2	18.0	14.9	25.0	15.9	0.023
**Kidney disease (yes)**	146	13.9	10.7	13.7	16.7	22.9	12.2	0.006
**Gastrointestinal disease (yes)**	430	41.0	35.0	41.0	36.2	51.4	53.0	<0.001
**Cancer (yes)**	165	15.7	15.1	12.2	16.1	17.1	18.9	0.58
**Self-rated health**								<0.001
Excellent	70	6.8	11.1	5.2	5.4	1.5	3.1	
Very good	341	33.3	39.4	26.7	32.3	26.5	29.8	
Good	340	33.2	30.7	42.2	35.9	33.1	29.8	
Fair/ Poor	272	26.6	18.9	25.9	26.3	39.0	37.3	
**No. of drugs**	1,048	4.5 ± 2.8	2.8 ± 1.9	5.2 ± 2.4	5.1 ± 2.6	6.5 ± 2.8	6.1 ± 2.8	<0.001
**Polypharmacy (yes)**	465	44.4	17.4	60.4	51.1	75.7	67.7	<0.001
**Hyper-Polypharmacy (yes)**	65	6.2	0.5	6.5	7.5	12.9	14.0	<0.001

*ApoE*, Apolipoprotein E; *BMI*, Body mass index; *BZDs*, Benzodiazepines; *CCBs*, Calcium channel blockers; *HMO*, Health maintenance organization; *NSAIDs*, Nonsteroidal anti-inflammatory drugs; *RAASi*, Renin angiotensin-aldosterone system inhibitors.

^a^The highest of the subject and his/ her spouse. Upper White collar includes scientific and academic professionals, senior managers, freelancers and technical professionals; lower White collar includes administrative and clerical workers; and Blue collar includes manual and skilled trade occupations, such as agriculture, fishing, craft, manufacturing, repair, construction, machine operation, transport, cleaning, packaging and general labor workers.

^b^ApoE ε2 group includes ε2/2 and ε2/3, ApoE ε3 group includes ε3/3, ApoE ε4 group includes ε4/4 and ε3/4, and ‘Other’ group includes the rare ε2/4 or missing observations.

^c^The number of chronic conditions reported in the study questionnaire and completed from diagnoses at hospitalizations. The chronic conditions of interest were cancer, cardiovascular disease, lung disease, joint diseases, kidney diseases, diabetes, eye disease, gastrointestinal disease, and hypertension.

### High-risk drug patterns and mortality

Overall, 64.8% of participants died over a median follow-up of 14.3 years (13,591 person-years). The number of events and unadjusted event rates for all-cause and non-cancer mortality, both overall and by cluster, are presented in Table 2.

**Table 2:** Unadjusted mortality rates per 1,000 person-years of follow up, overall and by cluster.

High-risk drug pattern	Person-years	All-cause mortality			Non-cancer mortality		
		Events	Rate	95% CI	Events	Rate	95% CI
Cluster 1, *None*	6228	240	38.5	33.7-43.4	180	28.9	24.7-33.1
Cluster 2, *CCBs*	1691	95	56.2	44.9-67.5	83	49.1	38.5-59.6
Cluster 3, *RAASi*	2284	113	49.5	40.4-58.6	102	44.7	36.0-53.3
Cluster 4, *Diuretics*	1472	111	75.4	61.4-89.4	96	65.2	52.2-78.2
Cluster 5, *BZDs*	1916	120	62.6	51.4-73.8	94	49.1	39.1-59.0
Overall	13,591	679	50.0	46.2-53.7	555	40.8	37.4-44.2

*BZDs*, Benzodiazepines; *CCBs*, Calcium channel blockers; *CI,* Confidence interval; *RAASi*, Renin angiotensin-aldosterone system inhibitors.

**Fig 2:**
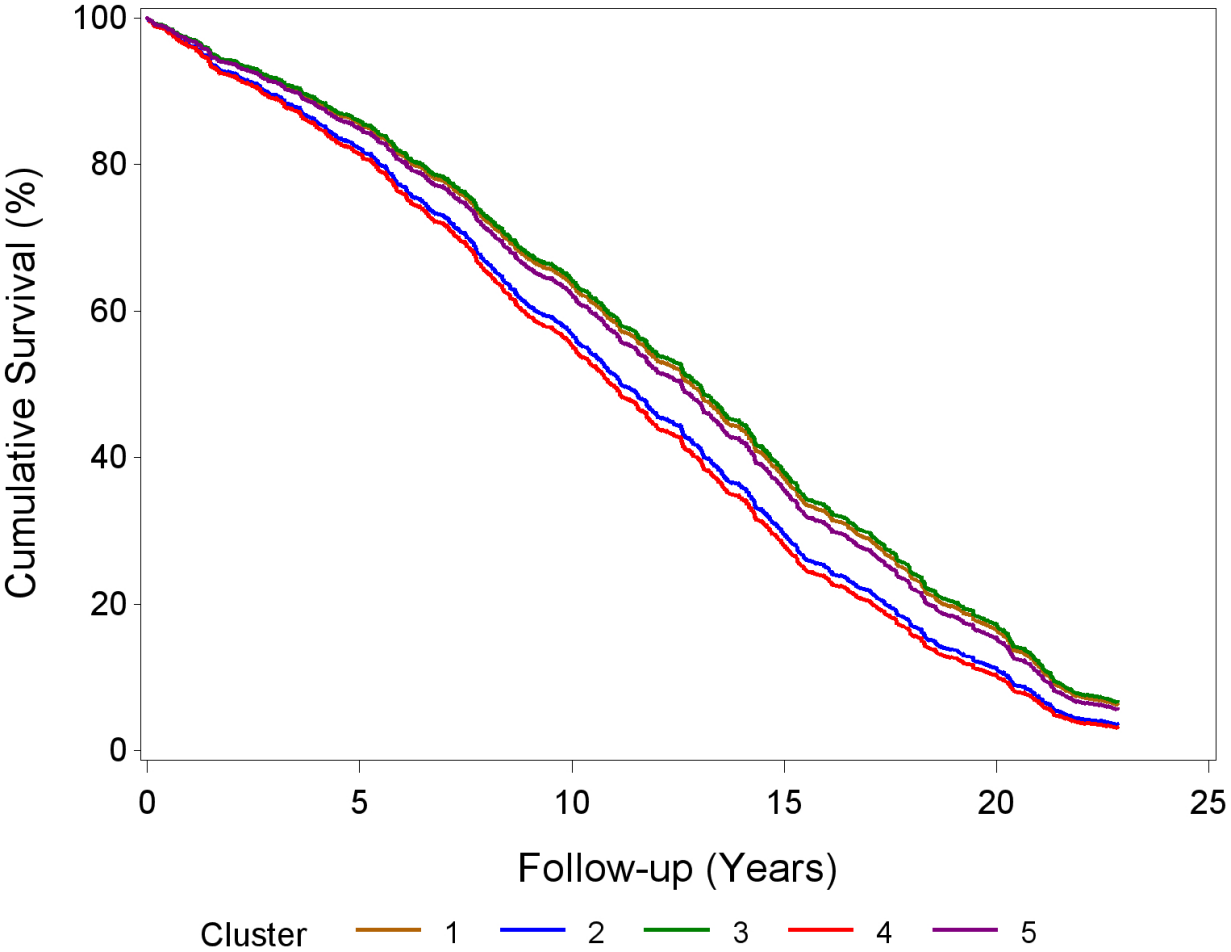
Survival curves for all-cause mortality according to high-risk drug patterns among community-dwelling older adults. Cox proportional hazard model was adjusted for: age, sex, polypharmacy, physical activity, BMI, smoking, ApoE genotype, self-rated health, diabetes, cardiovascular disease, cancer and count of remaining comorbidities. High-risk drug patterns: Cluster 1 = ‘None’ cluster (reference); Cluster 2 = ‘Calcium channel blockers (CCBs)’ cluster; Cluster 3 = ‘Renin-angiotensin-aldosterone system (RAAS) inhibitors’ cluster; Cluster 4 = ‘Diuretics’ cluster. Only Cluster 4 significantly differed from Cluster 1 (HR = 1.33, 95%CI: 1.03-1.72); Cluster 5 = ‘Benzodiazepines (BZDs)’ cluster.

The ‘None’ cluster, which was the largest and had no prevalent high-risk drug, was set as the reference category. In the unadjusted Cox regression model (see Table 3), compared with the ‘None’ cluster, each of the other drug patterns was associated with increased all-cause mortality risk (HRs range 1.35–2.19). Interestingly, adjustment for age and sex alone substantially attenuated the observed associations (S3 Table). In the fully adjusted model, considering sociodemographic, lifestyle, genetic, subjective and objective health measures, only the ‘Diuretics’ cluster was significantly associated with increased mortality risk (HR = 1.33, 95%CI: 1.03–1.72). The ‘CCBs’ cluster had an HR of 1.27 (95%CI: 0.98–1.64), whereas the other clusters presented no increased risk (HRs = 0.98 and 1.05 for the ‘RAAS inhibitors’ and ‘BZDs’ clusters, respectively; Table 3 and S4 Table). The adjusted survival curves for the five high-risk drug patterns are presented in Fig 2.

**Table 3:** Association between high-risk drug patterns and up to 23-year mortality among community-dwelling older adults.

High-risk drug pattern	All-cause mortality		Non-cancer mortality^b^
	Unadjusted	Adjusted^a^	
	HR (95% CI)	HR (95% CI)	HR (95% CI)
Cluster 1, *None*	ref.	ref.	ref.
Cluster 2, *CCBs*	**1.60 (1.26-2.03)**	1.27 (0.98-1.64)	**1.47 (1.11-1.94)**
Cluster 3, *RAASi*	**1.35 (1.08-1.69)**	0.98 (0.77-1.24)	1.25 (0.97-1.61)
Cluster 4, *Diuretics*	**2.19 (1.75-2.75)**	**1.33 (1.03-1.72)**	**1.41 (1.03-1.93)**
Cluster 5, *BZDs*	**1.76 (1.41-2.20)**	1.05 (0.82-1.35)	1.08 (0.80-1.44)

*BZDs*, Benzodiazepines; *CCBs*, Calcium channel blockers; *CI,* Confidence interval; *HR,* Hazard ratio; *RAASi*, Renin angiotensin-aldosterone system inhibitors.

^a^Adjusted for age, sex, polypharmacy, physical activity, BMI, smoking status, ApoE genotype, self-rated health, diabetes, cardiovascular disease, cancer and count of remaining comorbidities.

^b^Adjusted for age, sex, polypharmacy, education, health maintenance organization, occupation, marital status, smoking, ApoE genotype, self-rated health, diabetes, cardiovascular disease, cancer and count of remaining comorbidities. Due to proportional hazards assumption violation, additionally adjusted for time interactions with cardiovascular disease, diabetes, ApoE and remaining comorbidities count variables.

In the competing risks analysis, both the ‘Diuretics’ and ‘CCBs’ clusters were significantly associated with increased non-cancer mortality risk (HR = 1.41 and 1.47, respectively), after adjusting for sociodemographic, lifestyle, genetic, and both subjective and objective health measures, and accounting for violations of the proportional hazards assumption (see Table 3 and S5 Table). The ‘RAAS inhibitors’ cluster was also associated with some increased risk, although to a lesser extent (HR = 1.25, 95%CI: 0.97–1.61), whereas the ‘BZDs’ cluster did not differ from the ‘None’ cluster.

Results remained similar across the sensitivity analyses (see Models 1–6 in Table 4).

**Table 4:** Sensitivity analyses for the association between high-risk drug patterns and up to 23-year all-cause mortality among community-dwelling older adults.

High-risk drugs patterns	Model 1^a^	Model 2^b^	Model 3^c^	Model 4^d^	Model 5^e^	Model 6^f^
	HR (95% CI)	HR (95% CI)	HR (95% CI)	HR (95% CI)	HR (95% CI)	HR (95% CI)
Cluster 1, *None*	ref.	ref.	ref.	ref.	ref.	ref.
Cluster 2, *CCBs*	1.33 (1.00-1.77)	1.27 (0.98-1.65)	1.28 (0.97-1.68)	1.26 (0.97-1.64)	1.26 (0.97-1.64)	1.24 (0.96-1.61)
Cluster 3, *RAASi*	0.99 (0.76-1.29)	0.99 (0.77-1.26)	0.87 (0.67-1.15)	0.96 (0.75-1.22)	0.97 (0.76-1.24)	0.96 (0.75-1.21)
Cluster 4, *Diuretics*	**1.34 (1.03-1.76)**	**1.37 (1.06-1.77)**	1.28 (0.97-1.68)	**1.33 (1.03-1.72)**	**1.32 (1.02−1.70)**	**1.32 (1.03−1.71)**
Cluster 5, *BZDs*	1.06 (0.82-1.38)	1.05 (0.82-1.35)	0.98 (0.75-1.28)	1.05 (0.82-1.35)	1.04 (0.81-1.34)	1.03 (0.80-1.32)

*BZDs*, Benzodiazepines; *CCBs*, Calcium channel blockers; *CI,* Confidence interval; *HR,* Hazard ratio; *RAASi*, Renin angiotensin-aldosterone system inhibitors.

^a^Accounting for individual cardiovascular-related diseases: Ischemic heart disease, stroke, other heart disease and hypertension. Also adjusted for age, sex, polypharmacy, physical activity, BMI, smoking status, ApoE genotype, self-rated health, diabetes, cancer, count of remaining comorbidities, and time interactions with age and other heart disease variables.

^b^Accounting for pack years of smoking and time since quitting instead of smoking status. Also adjusted for age, sex, polypharmacy, physical activity, BMI, ApoE genotype, self-rated health, diabetes, cardiovascular disease, cancer and count of remaining comorbidities.

^c^Covering only pre-COVID-19 period in Israel (truncated on 21Feb20). Adjusted for age, sex, polypharmacy, physical activity, BMI, smoking status, ApoE genotype, self-rated health, diabetes, cardiovascular disease, cancer, and count of remaining comorbidities.

^d^Accounting for birth cohort, in addition to participants’ age. Also adjusted for sex, polypharmacy, physical activity, BMI, smoking status, ApoE genotype, self-rated health, diabetes, cardiovascular disease, cancer and count of remaining comorbidities.

^e^Considering an alternative definition for polypharmacy. Also adjusted for age, sex, physical activity, smoking status, BMI, ApoE genotype, self-rated health, diabetes, cardiovascular disease, cancer and count of remaining comorbidities.

^f^Multiple imputation analysis. Adjusted for age, sex, polypharmacy, physical activity, BMI, smoking status, ApoE genotype, self-rated health, diabetes, cardiovascular disease, cancer, count of remaining comorbidities, and time interactions with age, BMI and physical activity.

## Discussion

Our study revealed five distinct high-risk drug patterns among community-dwelling older adults: (1) ‘None’ cluster (i.e., no dominant high-risk drug), (2) ‘CCBs’ cluster, (3) ‘RAAS inhibitors’ cluster, (4) ‘Diuretics’ cluster and (5) ‘BZDs’ cluster. The ‘Diuretics’ cluster represented the most complex patients (i.e., were older, had a higher number of comorbidities and a higher prevalence of polypharmacy), and was most consistently associated with increased mortality risk, for both all-cause and non-cancer mortality. This suggests that older adults taking diuretics, PPIs, antithrombotics and antipsychotics face increased mortality risk and warrant special attention. An increased risk, mainly for non-cancer mortality, was also observed for the ‘CCBs’ cluster, whereas the other clusters did not significantly differ from the ‘None’ cluster in any of the analyses. Findings from the competing risks analysis were directionally consistent with the Cox models, with slightly stronger effect estimates, supporting our assumption that cancer deaths are less likely to be influenced by drug use.

Several drug combinations could have contributed to the increased risk observed for the ‘Diuretics’ cluster. Over 57% of its participants were concurrently using a CCB. Although their concomitant use may be appropriate in cases requiring multiple agents or with an underlying cardiovascular disease, it nevertheless might exacerbate the risk of ADRs [48]. Moreover, in some cases this may result from an inappropriate CCB-diuretic prescribing cascade, which raises the risk of falls, acute kidney injury, and other serious adverse events [18,49]. Older hypertensive patients should undergo regular medication review to ensure optimal treatment. Clinicians should be aware of nonpharmacologic strategies for CCB-induced edema.

Over half of participants in the ‘Diuretics’ cluster also used NSAIDs, which can reduce diuretic efficacy. Additionally, drug interactions can exacerbate NSAIDs-related ADRs, such as bleeding, coronary events and renal insufficiency [50], a concern given the high prevalence of kidney disease in this cluster. Toxicity related to the concomitant use of NSAIDs and antihypertensives may also have contributed to the increased non-cancer mortality risk seen in the ‘CCBs’ cluster [12,15].

The ‘Diuretics’ cluster had the highest prevalence of PPIs and antithrombotics. In addition to their individual risks (e.g., *Clostridium difficile* infection and fractures from prolonged PPI use [12], and increased thrombosis and bleeding risks with antithrombotics [51]), their combined use can cause harmful interactions and reduce antithrombotic efficacy [52]. Finally, this cluster had the highest antipsychotic use. While crucial for some conditions, their wide range of side effects [12] might reduce quality of life and impair medication adherence [53]. Patient preferences should guide antipsychotic choices.

Of note are the differences in sex distribution across clusters. Only the ‘RAAS inhibitors’ cluster had a majority of men. Moreover, Cluster 5, with the highest BZD and antidepressant use, had nearly two-thirds women. A similar pattern was observed by Huang et al. [23], where the cluster with the highest prevalence of RAAS inhibitors was the only one with a majority of men, and the ‘Mental health drugs’ cluster had 61.1% women. Future studies should explore sex-specific high-risk drug patterns in older people.

Interestingly, even after adjustment for socioeconomic, lifestyle, and health-related measures, ApoE genotype was significantly associated with a 1.5-fold increased mortality risk. ApoE genotype may affect both exposure (via predisposition to certain diseases [54,55]) and mortality risk [56]. Furthermore, pharmacogenomic/pharmacogenetic studies suggest a role for ApoE polymorphism in the inter-individual variability in drug responses, with ε4 carriers having poor responses to certain treatments [57,58] and higher ADR risks [58,59]. The mechanism may involve effects of the ApoE gene on liver drug metabolism [57]. Owing to limited statistical power, we could not explore possible interactions between ApoE genotype and drug patterns. Further research is needed to investigate ApoE as a biomarker for pharmacogenetic responses.

In addition, the lack of variation in education and occupation across medication clusters may reflect the influence of Israel’s universal health insurance system, which ensures access to essential medications and healthcare services for all citizens. However, not all medications are covered under the subsidized Health Basket, and many require co-payments. While certain protections exist for vulnerable populations, such as quarterly spending caps for chronically ill patients, welfare recipients and holocaust survivors, research has shown that pharmaceutical co-payments can still pose a substantial financial burden on the poor, elderly, and chronically ill, potentially impairing adherence [60]. Sociodemographic factors should be considered when analyzing medication use and health outcomes, even in a universal healthcare setting.

Finally, polypharmacy was not significantly associated with mortality risk in any of the analyses. This suggests that considering medication patterns may be more valuable in identifying high-risk patients than merely their count.

Several study limitations should be acknowledged. First, medication data were collected only at baseline and may not reflect current prescribing trends or changes over time affecting drug exposure or its association with mortality (such as shifts in population health behaviors). Participants may also have altered their drug use during follow-up due to health changes or adherence issues. The broad medication categories, encompassing one or more drug classes, partly addressed this limitation. We also conducted two sensitivity analyses to account for time-related factors (a potential cohort effect and possible pandemic-related changes in health-services utilization or health behaviors), both of which yielded consistent results, supporting the robustness of our findings. Second, although we adjusted for multiple health-related factors, we did not account for dosage or adherence, which could influence drug effects and interactions. Third, the sample size limited our ability to assess additional cause-specific mortality outcomes. Fourth, some self-reported measures (e.g., physical activity, smoking history) may be subject to recall bias. Fifth, our cohort included only Jewish older adults in Israel, which may limit generalizability, although participants were from diverse ethnic backgrounds. Sixth, survival bias due to informative censoring and/or left truncation is possible, as participants who died before their third interview were not included. This may have led to underestimating high-risk drug use in the general older population, but offered insights into the impact on relatively healthy older adults. Finally, our study was not designed to establish causal inference about specific drugs, but rather to identify profiles of high-risk patients, defined by their patterns of drug use, who may benefit from interventions targeting their drug regimens. We therefore considered only potentially inappropriate or high-risk medications, investigated data-driven patterns rather than pre-defined combinations or individual drugs, and examined their associations with mortality while adjusting for a broad range of potential confounders, including sociodemographic and health-related characteristics. These included individual conditions known as major risk factors of mortality, a count of additional chronic conditions, and self-rated health, a measure consistently associated with mortality and reflective of severity of both diagnosed and undiagnosed diseases [61].

The strengths of this study include its population-based, ethnically diverse sample of community-dwelling older men and women, the high-quality data, recorded at a state of health for research purposes, along with the availability of information on OTC drugs, which enabled us to identify ‘real-world’ high-risk drug patterns. Furthermore, the complete long-term follow-up enabled the accumulation of ADR-related downstream clinical outcomes, ultimately leading to premature mortality. Finally, our study employs a patient-centered, advanced approach, combining hierarchical clustering with survival analysis, to identify clinically meaningful usage patterns of common high-risk medications among community-dwelling older adults.

## Conclusion

In this prospective cohort study of community-dwelling older adults, five clusters were identified. The concurrent use of diuretics, PPIs, antithrombotics and antipsychotics (‘Diuretics’ cluster) was most consistently associated with overall and non-cancer mortality. The ‘CCBs’ cluster showed a greater risk mainly for non-cancer mortality. Specific high-risk drug combinations, such as NSAIDs with antihypertensives, may have further contributed to the observed increased mortality risk. As patterns of drug regimens reflect disease burden and treatment complexity, may confer additional risks (including through drug-disease interactions), and are modifiable, they may serve as a practical tool to support risk stratification efforts and guide early interventions for vulnerable older patients (e.g., frequent medication reviews, dosage adjustments and closer monitoring of specific biomarkers/ADRs). To advance more personalized care, future research can harness artificial intelligence to characterize high-risk drug patterns across diverse subpopulations.

## Supporting information

S1 Text Detailed information on methodology. (DOCX)

S1 Table High-risk drug categories used by 1,048 community-dwelling older adults. (DOCX)

S2 Table Selected baseline characteristics of study participants by completeness of data. (DOCX)

S3 Table Cox proportional hazard models for the association between high-risk drug patterns and all-cause mortality, with incremental adjustment. (DOCX)

S4 Table Fully adjusted Cox proportional hazard model for the association between high-risk drug patterns and all-cause mortality. (DOCX)

S5 Table Adjusted Fine and Gray subdistribution hazard model for the association between high-risk drug patterns and non-cancer mortality. (DOCX)

S1 Fig Dendrogram of cluster analysis in 1,048 community-dwelling older adults. (PDF)

S2 Fig Elbow method plot for determining the optimal number of clusters. (PDF)
