# Supplementary material for: Associations of high-risk drug patterns with mortality among community-dwelling older adults: A 23-year prospective cohort study
Source: PLoS One. 2025 Sep 11;20(9):e0332210. doi: 10.1371/journal.pone.0332210 (PMC12425332; doi:10.1371/journal.pone.0332210)
Supplement: S1 Text — (DOCX) [file pone.0332210.s001.docx]

**S1 Text: Detailed methodology for sensitivity analyses**

Six sensitivity analyses were employed:

(1) Adjusting for individual cardiovascular-related diseases, i.e. ischemic heart disease, stroke, other heart disease and hypertension.

(2) Adjusting for pack years of smoking instead of smoking status. The number of cigarettes smoked per day was divided by 20 and multiplied by the total number of years of smoking. Smoking was then categorized into 5 groups, according to the median of pack-years and time since quitting: never smokers, <23 pack years and quit 15+ years ago, <23 pack years and quit <15 years ago/current smokers, 23+ pack years and quit 15+ years ago, 23+ pack years and quit <15 years ago/current smokers.

(3) Truncating follow-up time at 21Feb20, the date of Israel's first diagnosed Covid-19 case. Considering only the pre-Covid-19 period was aimed to address potential bias resulting from pandemic-related changes in health-services utilization or health behaviors.

(4) Adjusting for birth cohort (1912-21, 1922-32, 1932-41) in addition to participants’ age. This was meant to address time-related factors affecting drug exposure or its association with mortality (e.g. prescription trends and changes in health-behaviors of the population).

(5) Considering an alternative definition for polypharmacy, excluding vitamins and dietary supplements. For this definition, the following ATC codes were not included in the drug count: A11, A12, A13, V06, C10AX06.

(6) Multiple imputation (MI) analysis. MI was performed using the fully conditional specification (FCS) method with the MNAR (Missing Not At Random) statement. The imputation process included 10 iterations and applied control-based pattern imputation, which assumes that after drop-out, the unobserved values in the experimental group follow the path of observed values in the control group. Results were pooled using the MIANALYZE procedure.
