# Supplementary material for: Associations of high-risk drug patterns with mortality among community-dwelling older adults: A 23-year prospective cohort study
Source: PLoS One. 2025 Sep 11;20(9):e0332210. doi: 10.1371/journal.pone.0332210 (PMC12425332; doi:10.1371/journal.pone.0332210)
Supplement: S1 Table — (DOCX) [file pone.0332210.s002.docx]

S1 Table: High-risk drug categories used by 1,048 community-dwelling older adults

| **High-risk drug category** | **Included drugs** | n | **%** |
| --- | --- | --- | --- |
| NSAIDs | NSAIDs, including aspirin | *451* | 43.0 |
| RAAS inhibitors | Angiotensin-converting-enzyme inhibitors (ACEIs)  Angiotensin II receptor blockers (ARBs) Renin inhibitors | *327* | 31.2 |
| CCBs | Both dihydropyridines and non-dihydropyridines | *262* | 25.0 |
| Diuretics | Thiazide-like diuretics  Loop diuretics  Potassium-sparing diuretics  Diuretics and Potassium-sparing agents combinations | *219* | 20.9 |
| BZDs | BZDs, BZD derivatives and non-BZD derivatives | *181* | 17.3 |
| Sulfonylureas , long acting | Glyburide | *90* | 8.6 |
| PPIs (unless for high-risk patients) | PPIs usage for >8 weeks | *70* | 6.7 |
| Antithrombotics | Anticoagulants Anti-platelets | *69* | 6.6 |
| Antidepressants | Tricyclic antidepressants (TCAs)  Selective serotonin reuptake inhibitors (SSRIs)  Serotonin and norepinephrine reuptake inhibitors (SNRIs) | *67* | 6.4 |
| Peripheral alpha‐1 blockers (Unless for benign prostatic hyperplasia) | Doxazosin and Terazosin (unless used to increase urinary flow in men with BPH) | *49* | 4.7 |
| Opioids | Opioid derivatives | *39* | 3.7 |
| Other CNS drugs^a^ | Epilepsy: anticonvulsants (without BZD derivatives)  Parkinson’s disease: carbidopa-levodopa  Acetylcholinesterase inhibitor: donepezil Dry mouth: pilocarpine | *28* | 2.7 |
| Antipsychotics | Atypical antipsychotics  Typical antipsychotics  Lithium and carbamazepine | *32* | 3.1 |
| Other anticholinergics^a^ | Urinary incontinence: Oxybutynin, Tolterodine  Parkinson’s disease: Trihexyphenidyl  Antiarrhythmic: Disopyramide  Antihistamines (first generation): Hydroxyzine, Clidinium‐chlordiazepoxide Antispasmodics: Dicyclomine | *26* | 2.5 |
| Estrogens^b^ | Estrogens | *19* | 1.8 |
| Steroids^b^ | Prednisone | *15* | 1.4 |
| Amiodarone^b^ | Unless patient has heart failure | *15* | 1.4 |
| Digoxin^b^ | Unless ≤0.125 mg per day | *8* | 0.8 |
| Antiemetics^b^ | Metoclopramide (unless for gastroparesis with duration of use < 12 weeks) | *2* | 0.2 |

*NSAIDs*, Non-steroidal anti-inflammatory drugs; *RAASi*, Renin angiotensin-aldosterone system inhibitors; *CCBs*, Calcium channel blockers; *BZDs*, Benzodiazepines; *PPIs*, Proton-pump inhibitors; BPH, Benign prostate hyperplasia; CNS, Central nervous system.

^a^ Remaining drugs not included in other categories

^b^ Were not included in the study due to frequency < 2%
