# Supplementary material for: Associations of high-risk drug patterns with mortality among community-dwelling older adults: A 23-year prospective cohort study
Source: PLoS One. 2025 Sep 11;20(9):e0332210. doi: 10.1371/journal.pone.0332210 (PMC12425332; doi:10.1371/journal.pone.0332210)
Supplement: S2 Table — (DOCX) [file pone.0332210.s003.docx]

S2 Table: Selected baseline characteristics of study participants by completeness of data

|  | **No missing values (*n*=767)** | **Any missing values (*n*=281)** | **P-value** |
| --- | --- | --- | --- |
| **Age (years),** mean±SD | 72.5±7.0 | 75.4±7.7 | <0.001 |
| Sex, % |  |  | <0.001 |
| Men | 47.7 | 34.2 |  |
| Women | 52.3 | 65.8 |  |
| **Education (years),** median [Q1,Q3] | 11 [8, 12] | 8 [5, 12] | <0.001 |
| **Smoking,** % |  |  | 0.14 |
| Never | 57.2 | 64.1 |  |
| Past | 33.5 | 28.1 |  |
| Current | 9.3 | 7.8 |  |
| **Occupation^a^, %** |  |  | 0.002 |
| Upper White collar | 40.9 | 32.3 |  |
| Lower White collar | 19.8 | 16.1 |  |
| Blue collar | 39.2 | 51.6 |  |
| **Self-rated health** |  |  | <0.001 |
| Excellent | 7.6 | 4.7 |  |
| Very good | 34.4 | 30.1 |  |
| Good | 35.3 | 27.0 |  |
| Fair/ Poor | 22.7 | 38.3 |  |
| **Any sports related physical,** % **activity (yes),** % | 52.3 | 37.4 | <0.001 |
| **No. of comorbidities^b^,** mean±SD | 2.8±1.7 | 3.1±1.9 | 0.016 |
| **No. of drugs,** mean±SD | 4.5±2.8 | 4.5±2.9 | 0.88 |

^a^ The highest of the subject and his/ her spouse. Upper White collar includes scientific and academic professionals, senior managers, freelancers and technical professionals; lower White collar includes administrative and clerical workers; and Blue collar includes manual and skilled trade occupations, such as agriculture, fishing, craft, manufacturing, repair, construction, machine operation, transport, cleaning, packaging and general labor workers.

^b^ The number of chronic conditions reported in the study questionnaire and completed from diagnoses at hospitalizations. The chronic conditions of interest were cancer, cardiovascular disease, lung disease, joint diseases, kidney diseases, diabetes, eye disease, gastrointestinal disease, and hypertension.
