# Supplementary material for: Associations of high-risk drug patterns with mortality among community-dwelling older adults: A 23-year prospective cohort study
Source: PLoS One. 2025 Sep 11;20(9):e0332210. doi: 10.1371/journal.pone.0332210 (PMC12425332; doi:10.1371/journal.pone.0332210)
Supplement: S3 Table — (DOCX) [file pone.0332210.s004.docx]

S3 Table: Cox proportional hazard models for the association between high-risk drug patterns and all-cause mortality, with incremental adjustment

| **Covariates** | **High-risk drugs clusters** | **HR (95% CI)** | **P-value** |
| --- | --- | --- | --- |
| None | Cluster 1 (None) | ref. | **<0.001** |
|  | Cluster 2 (CCBs) | **1.60 (1.26-2.03)** |  |
|  | Cluster 3 (RAASi) | **1.35 (1.08-1.69)** |  |
|  | Cluster 4 (Diuretics) | **2.19 (1.75-2.75)** |  |
|  | Cluster 5 (BZDs) | **1.76 (1.41-2.20)** |  |
| Demographics | Cluster 1 (None) | ref. | **<0.001** |
| *(sex, log(age))* | Cluster 2 (CCBs) | **1.40 (1.10-1.78)** |  |
|  | Cluster 3 (RAASi) | 1.18 (0.94-1.48) |  |
|  | Cluster 4 (Diuretics) | **1.56 (1.24-1.97)** |  |
|  | Cluster 5 (BZDs) | **1.37 (1.10-1.71)** |  |
| + Health behaviors  *(physical activity, smoking status, BMI)* | Cluster 1 (None) | ref. | **0.002** |
|  | Cluster 2 (CCBs) | **1.42 (1.11-1.80)** |  |
|  | Cluster 3 (RAASi) | 1.12 (0.89-1.41) |  |
|  | Cluster 4 (Diuretics) | **1.54 (1.22-1.95)** |  |
|  | Cluster 5 (BZDs) | **1.27 (1.01-1.59)** |  |
| + Subjective health measure | Cluster 1 (None) | ref. | **0.005** |
| *(self-rated health)* | Cluster 2 (CCBs) | **1.47 (1.15-1.88)** |  |
|  | Cluster 3 (RAASi) | 1.13 (0.90-1.43) |  |
|  | Cluster 4 (Diuretics) | **1.47 (1.16-1.86)** |  |
|  | Cluster 5 (BZDs) | 1.24 (0.98-1.56) |  |
| + Genetics | Cluster 1 (None) | ref. | **0.004** |
| *(ApoE genotype)* | Cluster 2 (CCBs) | **1.47 (1.14-1.88)** |  |
|  | Cluster 3 (RAASi) | 1.12 (0.89-1.41) |  |
|  | Cluster 4 (Diuretics) | **1.48 (1.17-1.88)** |  |
|  | Cluster 5 (BZDs) | 1.24 (0.98-1.56) |  |
| + Polypharmacy | Cluster 1 (None) | ref. | **0.043** |
|  | Cluster 2 (CCBs) | **1.39 (1.07-1.79)** |  |
|  | Cluster 3 (RAASi) | 1.07 (0.84-1.35) |  |
|  | Cluster 4 (Diuretics) | **1.37 (1.07-1.77)** |  |
|  | Cluster 5 (BZDs) | 1.15 (0.90-1.47) |  |
| + Baseline individual health conditions  *( Diabetes, cardiovascular disease, cancer)* | Cluster 1 (None) | ref. | 0.052 |
|  | Cluster 2 (CCBs) | **1.30 (1.00-1.68)** |  |
|  | Cluster 3 (RAASi) | 1.00 (0.78-1.27) |  |
|  | Cluster 4 (Diuretics) | **1.37 (1.06-1.76)** |  |
|  | Cluster 5 (BZDs) | 1.09 (0.85-1.39) |  |
| + Count of remaining baseline comorbidities | Cluster 1 (None) | ref. | 0.076 |
|  | Cluster 2 (CCBs) | 1.27 (0.98-1.64) |  |
|  | Cluster 3 (RAASi) | 0.98 (0.77-1.24) |  |
|  | Cluster 4 (Diuretics) | **1.33 (1.03-1.72)** |  |
|  | Cluster 5 (BZDs) | 1.05 (0.82-1.35) |  |

*HR,* Hazard ratio; *CI,* Confidence interval; *CCBs*, Calcium channel blockers; *NSAIDs*, Non-steroidal anti-inflammatory drugs; *RAASi*, Renin angiotensin-aldosterone system inhibitors; *BZDs*, Benzodiazepines.
