# Supplementary material for: Associations of high-risk drug patterns with mortality among community-dwelling older adults: A 23-year prospective cohort study
Source: PLoS One. 2025 Sep 11;20(9):e0332210. doi: 10.1371/journal.pone.0332210 (PMC12425332; doi:10.1371/journal.pone.0332210)
Supplement: S4 Table — (DOCX) [file pone.0332210.s005.docx]

S4 Table: Fully adjusted Cox proportional hazard model for the association between high-risk drug patterns and all-cause mortality

|  | **HR (95% CI)** | **P-value** |
| --- | --- | --- |
| High-risk drugs clusters |  | 0. 076 |
| Cluster 1 (None) | ref. |  |
| Cluster 2 (CCBs) | 1.27 (0.98-1.64) |  |
| Cluster 3 (RAASi) | 0.98 (0.77-1.24) |  |
| Cluster 4 (Diuretics) | **1.33 (1.03-1.72)** |  |
| Cluster 5 (BZDs) | 1.05 (0.82-1.35) |  |
| **Polypharmacy (yes)** | 0.98 (0.81-1.18) | 0.80 |
| **Sex** |  | <0.001 |
| Men | ref. |  |
| Women | 0.71 (0.59-0.84) |  |
| **Log (age)** (1% increment) | 1.08 (1.07-1.09) | <0.001 |
| **Any sports related physical activity (yes)** | 0.83 (0.70-0.98) | 0.030 |
| **BMI** **(kg/m^2^)** |  | 0.022 |
| Normal | ref. |  |
| Overweight | 0.74 (0.60-0.93) |  |
| Obese | 0.90 (0.72-1.14) |  |
| Unknown | 1.04 (0.77-1.42) |  |
| **Smoking** |  | 0.037 |
| Never | ref. |  |
| Past | 1.02 (0.85-1.23) |  |
| Current | 1.43 (1.08-1.89) |  |
| **ApoE genotype**^a^ |  | 0.003 |
| ApoE ε3 | ref. |  |
| ApoE ε2 | 0.96 (0.72-1.28) |  |
| ApoE ε4 | 1.54 (1.20-1.97) |  |
| Other/missing | 1.27 (1.00-1.61) |  |
| **Diabetes (yes)** | 1.42 (1.18-1.69) | <0.001 |
| **Cardiovascular disease** **(yes)** | 1.47 (1.24-1.76) | <0.001 |
| **Cancer (yes)** | 1.41 (1.15-1.72) | <0.001 |
| **No. of comorbidities^b^** (1-unit increment) | 1.07 (1.00-1.16) | 0.057 |
| **Self-rated health** |  | 0.021 |
| Excellent/ Very good | ref. |  |
| Good | 1.14 (0.94-1.39) |  |
| Fair/ Poor | 1.38 (1.10-1.74) |  |

*HR,* Hazard ratio; *CI,* Confidence interval; *CCBs*, Calcium channel blockers; *NSAIDs*, Non-steroidal anti-inflammatory drugs; *RAASi*, Renin angiotensin-aldosterone system inhibitors; *BZDs*, Benzodiazepines.

^a^ ApoE ε2 group includes ε2/2 and ε2/3, ApoE ε3 group includes ε3/3, ApoE ε4 group includes ε4/4 and ε3/4, and ‘Other’ group includes carriers of the rare ε2/4.

**^b^** Remaining comorbidities not individually adjusted for in the model.
