# Supplementary material for: Associations of high-risk drug patterns with mortality among community-dwelling older adults: A 23-year prospective cohort study
Source: PLoS One. 2025 Sep 11;20(9):e0332210. doi: 10.1371/journal.pone.0332210 (PMC12425332; doi:10.1371/journal.pone.0332210)
Supplement: S5 Table — (DOCX) [file pone.0332210.s006.docx]

**S5 Table: Adjusted Fine and Gray subdistribution hazard model for the association between high-risk drug patterns and non-cancer mortality^a^**

|  | **HR (95% CI)** | P-value |
| --- | --- | --- |
| High-risk drugs clusters |  | 0. 037 |
| Cluster 1 (None) | ref. |  |
| Cluster 2 (CCBs) | **1.47 (1.11-1.94)** |  |
| Cluster 3 (RAASi) | 1.25 (0.97-1.61) |  |
| Cluster 4 (Diuretics) | **1.41 (1.03-1.93)** |  |
| Cluster 5 (BZDs) | 1.08 (0.80-1.44) |  |
| **Polypharmacy (yes)** | 1.11 (0.90-1.37) | 0.353 |
| **Sex** |  | <0.001 |
| Men | ref. |  |
| Women | 0.67 (0.55-0.83) |  |
| Age |  | <0.001 |
| Lowest tertile (58-68) | ref. |  |
| Middle tertile (69-75) | 2.40 (1.84-3.12) |  |
| Upper tertile (76-94) | 5.02 (3.81-6.61) |  |
| **Education** (1-year increment) | 0.97 (0.95-0.99) | 0.013 |
| **Occupation^b^** |  | 0.011 |
| Blue collar | ref. |  |
| Lower White collar | 1.46 (1.13-1.87) |  |
| Upper White collar | 1.15 (0.90-1.47) |  |
| **Married (yes)** | 1.24 (1.02-1.51) | 0.031 |
| **Smoking** |  | 0.037 |
| Never | ref. |  |
| Past | 0.85 (0.69-1.04) |  |
| Current | 1.33 (0.95-1.86) |  |
| **ApoE genotype^c^** |  | 0.002 |
| ApoE ε3 | ref. |  |
| ApoE ε2 | 0.91 (0.65-1.29) |  |
| ApoE ε4 | 1.38 (1.06-1.79) |  |
| Other/missing | 2.00 (1.31-3.07) |  |
| **Diabetes (yes)** | 2.30 (1.59-3.33) | <0.001 |
| **Cardiovascular disease** **(yes)** | 2.32 (1.55-3.47) | <0.001 |
| **Cancer (yes)** | 0.82 (0.64-1.05) | 0.116 |
| **No. of comorbidities ^d^** (1-unit increment) | 1.23 (1.06-1.43) | 0.007 |
| **Self-rated health** |  | 0.009 |
| Excellent/ Very good | ref. |  |
| Good | 0.98 (0.78-1.22) |  |
| Fair/ Poor | 1.39 (1.08-1.80) |  |

*HR,* Hazard ratio; *CI,* Confidence interval; *CCBs*, Calcium channel blockers; *NSAIDs*, Non-steroidal anti-inflammatory drugs; *RAASi*, Renin angiotensin-aldosterone system inhibitors; *BZDs*, Benzodiazepines.

^a^ Also adjusted for health maintenance organization and time interactions with cardiovascular disease, diabetes, ApoE and remaining comorbidities count variables.

**^b^** The highest of the subject and his/ her spouse. Upper White collar includes scientific and academic professionals, senior managers, freelancers and technical professionals; lower White collar includes clerical workers.

**^c^** ApoE ε2 group includes ε2/2 and ε2/3, ApoE ε3 group includes ε3/3, ApoE ε4 group includes ε4/4 and ε3/4, and ‘Other’ group includes carriers of the rare ε2/4.

**^d^** Remaining comorbidities not individually adjusted for in the model.
