# Supplementary figures and images for: Associations of high-risk drug patterns with mortality among community-dwelling older adults: A 23-year prospective cohort study
Source: PLoS One. 2025 Sep 11;20(9):e0332210. doi: 10.1371/journal.pone.0332210 (PMC12425332; doi:10.1371/journal.pone.0332210)

**S1 Fig: Dendrogram of cluster analysis in 1,048 community-dwelling older adults**

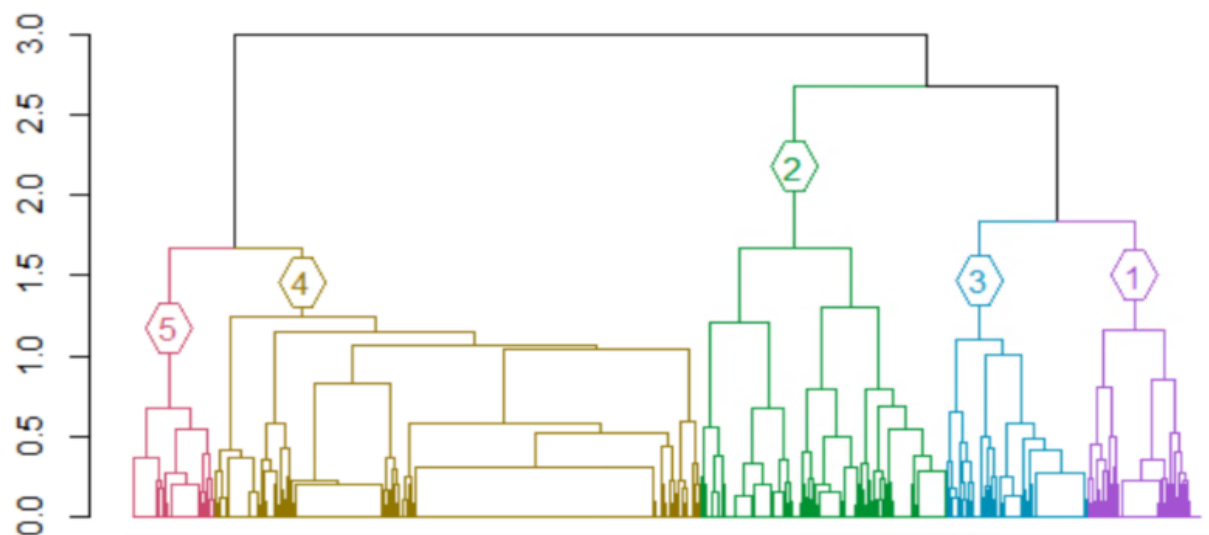

Supplement: S1 Fig — (PDF) [file pone.0332210.s007.pdf]
