# Supplementary material for: Associations of high-risk drug patterns with mortality among community-dwelling older adults: A 23-year prospective cohort study
Source: PLoS One. 2025 Sep 11;20(9):e0332210. doi: 10.1371/journal.pone.0332210 (PMC12425332; doi:10.1371/journal.pone.0332210)
Supplement: S2 Fig — (PDF) [file pone.0332210.s008.pdf]

S2 Fig: Elbow method plot for determining the optimal number of clusters.

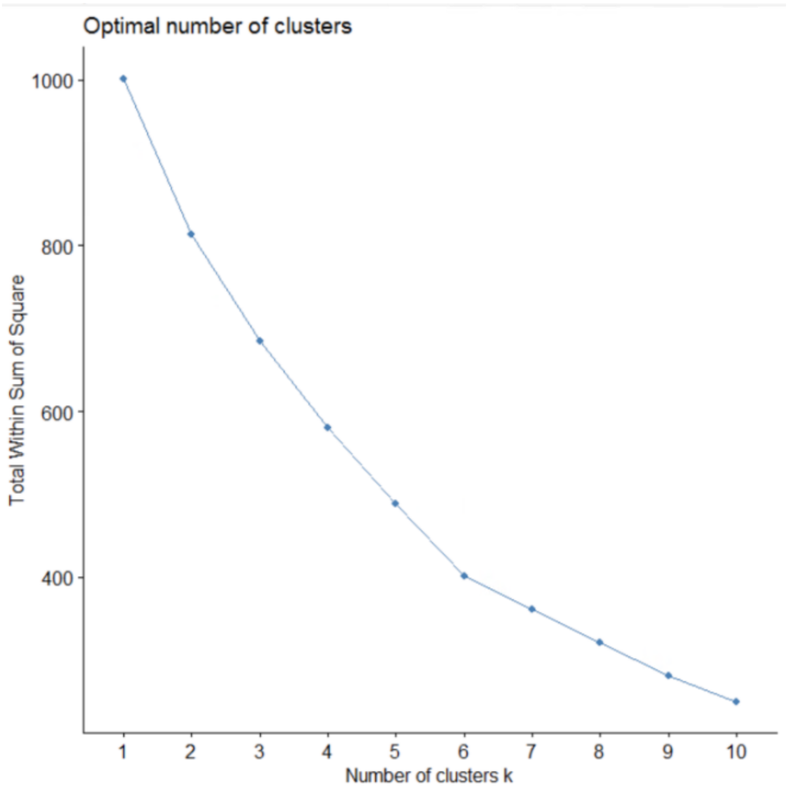

The Elbow plot suggests six clusters as optimal. However, five clusters were selected to ensure adequate statistical power (i.e., sufficient sample size within each cluster for analysis).
